# Does the Brain Drain Effect Really Exist? A Meta-Analysis

**DOI:** 10.3390/bs13090751

**Published:** 2023-09-11

**Authors:** Tobias Böttger, Michael Poschik, Klaus Zierer

**Affiliations:** Philosophical-Social Sciences Faculty, University of Augsburg, 86159 Augsburg, Germany; tobias.boettger@phil.uni-augsburg.de (T.B.); michael.poschik@uni-a.de (M.P.)

**Keywords:** brain drain, smartphone, attention, cognitive performance

## Abstract

Smartphones have become an indispensable part of everyday life. Given the current debate about the use of smartphones in classrooms and schools, it seems appropriate to examine their effects on aspects of cognitive performance in more detail. Ward and colleagues not only demonstrated the negative effect of smartphones on cognitive performance but also showed that the mere presence of these devices can have this effect—this is known as the Brain Drain effect. In the present article, a meta-analytic approach was adopted in order to verify these findings. Here we show a significant overall negative effect of smartphone use and presence. In a database search we identified 22 studies with a total of 43 relevant effects that could be assigned to the categories “memory”, “attention”, and “general cognitive performance”. A subgroup analysis suggests that not all cognitive domains are equally affected by the negative effect of smartphones. The heterogeneity of the effects reinforces this finding. The nationality of the test subjects or the origin of the studies was identified as a further key variable. Our findings also indicate that the distracting effect of smartphones varies on the area studies and further research is necessary. In view of the present research results, it seems important that people in general, and especially children and adolescents in schools and classrooms, learn how to deal with the distracting potential of smartphones.

## 1. Introduction

Smartphones are a part of most people’s lives, and increasingly so for children and young people. The devices are also being used more and more frequently in educational institutions. According to the Bavarian State Ministry of Education and Cultural Affairs, smartphones are used for teaching purposes (e.g., quizzes, digital learning environment, online research). Permission to use the digital devices privately is also being discussed [1]. However, there are countries that have completely banned smartphones from schools after years of testing. In France, smartphone use has been banned in elementary schools and colleges since September 2018 [2]. It is debatable whether the constant presence of this kind of media does more harm than good. Studies such as that by Ward and colleagues indicate that the mere presence of smartphones has negative effects on learning performance and attention [3]. Their study named this phenomenon the Brain Drain effect and it has led to a great deal of research activity in this area, with mixed results [4]. Thus, the question arises whether the Brain Drain effect really exists. Here we try to answer this question using meta-analytical techniques and deliberately choose a narrow focus. Based on the findings of the Brain Drain study, we examined studies that report effects of smartphones (tablets, smartwatches, and the like are not included in this meta-analysis) on cognitive processes. We focused on domains of cognitive performance that could be distinguished on the basis of the primary studies found.

Effects on the functioning of working memory seem to be at the centre of the impact of smartphone use on cognitive capacities. Study results indicate that the duration of smartphone use correlates with the availability of working memory [5]. On the one hand, the partially automatic control of attentional processes helps maintain attention, but on the other hand, it can be detrimental to cognitive performance if a stimulus (e.g., the receipt of an important message) is perceived as relevant to one’s goals but is not relevant to the current task [6]. General cognitive performance includes test scores to be completed in, for example, mathematics, native language, or science. This shows why smartphones have a distracting effect even if they are not being used but are in spatial proximity during the completion of a task. Smartphones can also disrupt focused attention when the user is trying to ignore them [7]. Interfering with automatic attentional processes demands attentional resources. Therefore, performance on tasks that rely on these capacities may also decline when attention is not consciously paid to the phone, but the mere presence or absence of the smartphone has an effect [8]. In light of the fact that Ward et al. (2017) [3] also investigated these forms of distraction, studies that refer to the mere presence of the smartphone are also relevant for the present study.

The country of origin of the studies or the nationality of the subjects examined in studies seems to play a role regarding the Brain Drain effect. This finding is supported by a study in which the distracting effect of smartphones was investigated. In this study, Asian students were able to concentrate better without smartphones than European students [9]. For this reason, the region of the studies is given an attention in the present meta-analysis.

## 2. Materials and Methods

**Figure 1:**
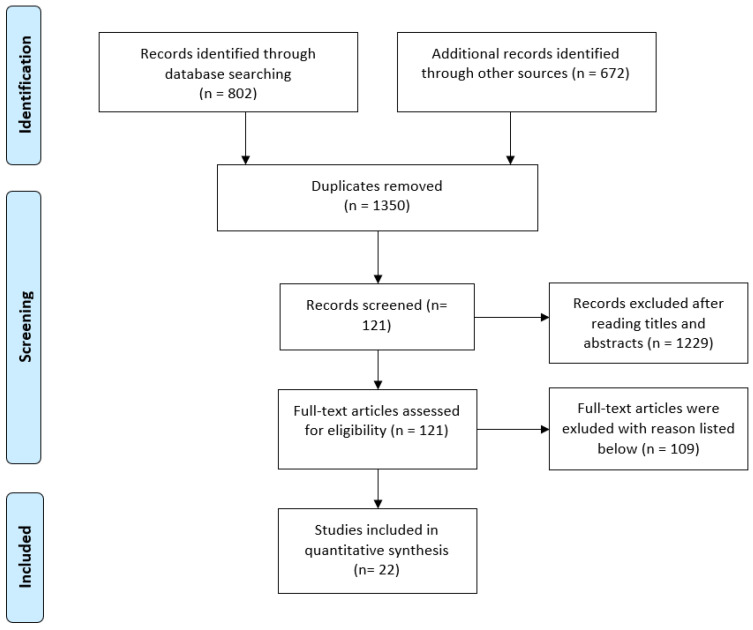
Study selection process.

The meta-analysis follows the PRISMA statement. A prerequisite for inclusion in this meta-analysis was the report of a measure of the effect of smartphones (e.g., means and standard deviations) on cognitive performance. Accordingly, the focus of the search was on those studies selected that were methodologically similar to the study design of the Ward et al. (2017) [3] review and aimed to confirm, or refute, the Brain Drain effect. We searched the databases PSYNDEX, Web of Science, and ResearchGate using the search pattern “(phone OR smartphone OR cellphone OR nomophobia) AND (attention OR awareness OR distraction OR vigilance OR concentration OR memor* OR concentrat* OR perform* OR “cognitive capacity” OR “Brain Drain” OR mere)”. This resulted in 802 hits. In addition, we searched the study by Ward et al. (2017) [3] using the “cited by” function of the Google Scholar search engine, which resulted in an additional 672 hits. The study search was completed on 10 December 2022. Subsequently, we eliminated 94 duplicates. We screened the remaining 1350 studies and narrowed them down to 121 after reading the abstracts. Only studies that employed quantitative-empirical approaches, investigating the impact of smartphones on cognitive performance and thereby establishing a direct connection to the Brain Drain effect, were included. Studies that had a qualitative-empirical design, lacked a connection to the Brain Drain study, or focused on other digital devices such as tablets and smartwatches were excluded. Finally, a full text analysis led to the exclusion of a further 109 studies due to a lack of fit between the research question and the research design. Finally, 22 studies met all search criteria (Figure 1).

Some studies have reported effects of smartphones or their presence that were mediated by the anxiety disorder nomophobia [9]. These studies also provide findings regarding the effects of smartphones on cognitive performance. Studies on smartphone-related addiction [10], the effects of electromagnetic fields on the human brain and its functioning [11], or effects on social skills and emotions [12] or the like, were not included. Some studies have reported multiple effects of smartphones or their presence on various cognitive functions (e.g., attention, fluid intelligence). Consequently, the number of effects (*n* = 43) exceeds the number of selected studies (*n* = 22) (Appendix A).

We performed all calculations using the Open Meta Analyst program (http://www.cebm.brown.edu/openmeta/#, accessed on 8 September 2023). Information on effect sizes and standard deviations was taken from the primary studies listed in Appendix A and entered into the calculation program.

## 3. Results

**Figure 2:**
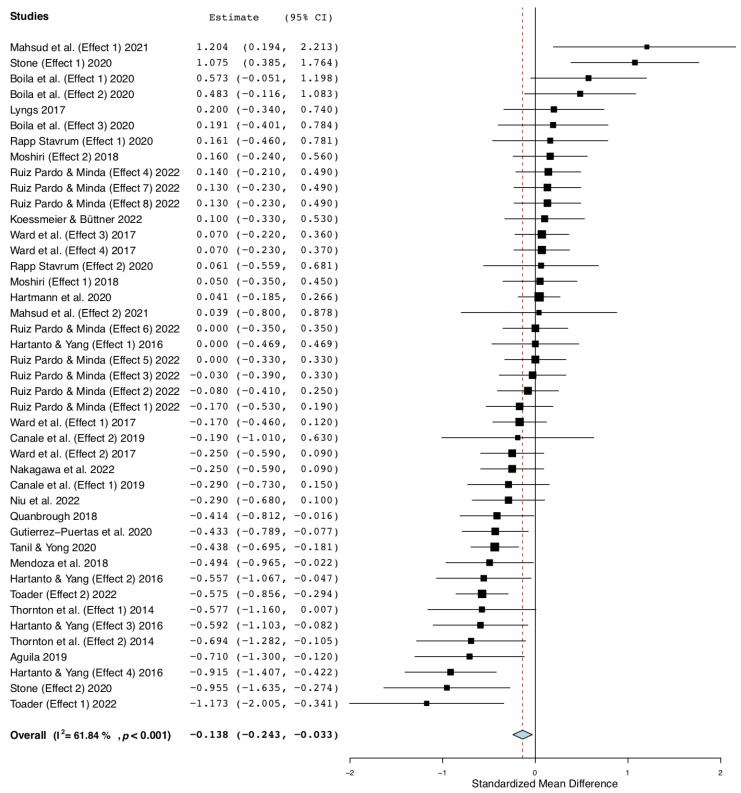
Overall effect [3,8,9,14,15,16,17,18,19,20,21,22,23,24,25,26,27,28,29,30,31].

Hedge’s *g* was selected as the effect size measure. The sample sizes across studies vary and, at times, are small. By utilizing the small sample bias correction, it becomes feasible to address potential biases in small samples, consequently yielding a more consistent and precise estimation of effect sizes. We calculated the pooled effect size using a continuous random effects model applying the DerSimonian and Laird method [13]. The pooled effect across all studies was *g* = −0.14, with a 95% confidence interval (CI) of −0.24 to −0.03, at *p* < 0.05 (Figure 2). The included effects show a significant heterogeneity (*Q* (df = 42) = 110.06, *I*^2^ = 61.84, *p* < 0.001).

**Figure 3:**
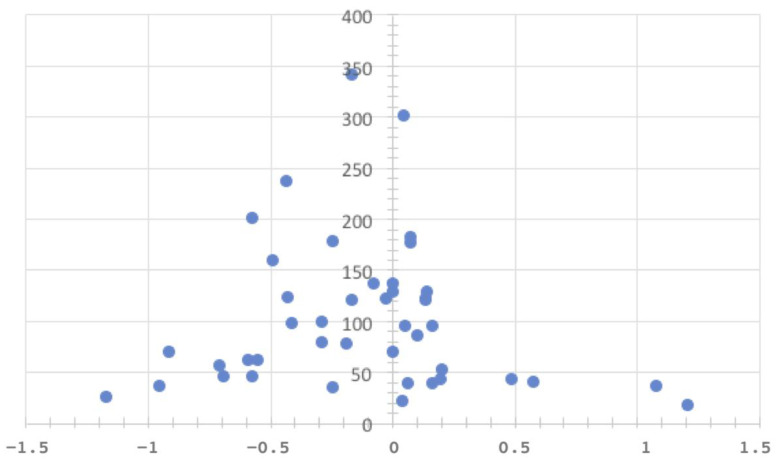
Effect sizes and sample size.

An examination of the effect sizes as a function of the underlying sample size reveals that the largest and smallest effects in each case were found among relatively small study groups. At the same time, the majority of positive effects were detected in comparatively small samples, while negative effects were also reported in samples that tended to be larger. The funnel plot does not indicate a possible bias or possible outliers, as we also found and took into account studies in which low effect sizes were reported for a small number of cases (Figure 3). In addition to the visual inspection of the funnel plot, which does not display any conspicuous indications of systematic bias, the False Discovery Rate (FDR) was controlled using the method proposed by Benjamini and Hochberg. Applying the FDR correction to the employed statistical tests helps mitigate the risk of random discoveries in the examination of multiple hypotheses [32,33].

The studies included in the present meta-analysis examined various aspects of cognitive performance. In terms of content, it was possible to distinguish the areas of “memory”, “attention”, and “general cognitive performance” (Appendix A). In the course of subgroup analyses, we examined these factors in more detail. Only the pooled effect of “memory” was significant (*g* = −0.23, 95% CI = −0.36; −0.10, *p* < 0.001). The “attention” effects (*g* = −0.07, 95% CI = −0.21; 0.06, *p* = 0.29) and “general cognitive performance” effects (*g* = 0.10, 95% CI = −0.52; 0.72, *p* = 0.76) were not. Despite the distinction of subgroups, significant heterogeneity was evident when we examined all three factors, just as it was when we calculated the overall effect (Table 1).

To determine possible causes for heterogeneity, we conducted another subgroup analysis in which the effects were grouped on the basis of the region from which the study originated. This approach is based on the findings of Mahsud et al. (2021), in which Asian subjects were influenced by smartphones to a different extent than European subjects [9]. A significant pooled effect could only be determined for the studies from the Asian region (*g* = −0.39, 95% CI = −0.57; −0.21, *p* < 0.001). The effects determined for the European (*g* = −0.20, 95% CI = −0.46; 0.06, *p* = 0.12) and North American (*g* = −0.03, 95% CI = −0.15; 0.09, *p* = 0.60) regions did not reach the significance threshold. The effects from the Asian region showed no signs of heterogeneity. In the case of North America and Europe, the heterogeneity was significant (Table 2).

## 4. Discussion

The present meta-analysis essentially confirms the Brain Drain effect, which is negative regarding cognitive performance. The results vary with respect to the different domains that were examined here. They are strongest for memory and lower for attention and general cognitive performance. Some studies showed that both smartphone use and the presence of the device result in negative effects on aspects of cognitive performance. However, not all studies reached this conclusion [16]. Consequently, heterogeneous findings may also emerge with respect to smartphone use and its presence. Moreover, variables such as smartphone dependence, which are discussed in some primary studies, may have a crucial influence on the distracting effect of these devices [9]. Moreover, this current meta-analysis encompassed studies employing various instruments to examine diverse cognitive performance indicators. While we took into account a range of results, it is important to acknowledge the presence of some variability in the observed effects. Due to the number of available effects, survey instruments, and possible implications, it seems obvious that subgroups that are more homogeneous still show signs of heterogeneity.

The present meta-analysis includes studies and effects from different countries. As the nationality of the subjects may cause heterogeneity, we performed a subgroup analysis that took into account the regions in which the respective surveys were conducted. It showed that the overall negative effect of smartphones was more pronounced and significant in the Asian region. Unfortunately, we did not find a satisfactory explanation for this result. It seems obvious that more research is needed both to provide further evidence for the Brain Drain effect and to further explain existing differences in terms of domain and region. Due to the small number of primary studies, further calculations, for example based on different measurement instruments or subjects, was not possible at this stage, but may be of interest in the future.

Certain limitations need to be addressed: As the inclusion criteria for the selection of studies based on the Brain Drain study, a total of 22 studies were included in the review, making an expansion of the data pool desirable in the coming years. Another limiting factor was the nature of outcome reporting in the various studies. Means and standard errors were not always available. In addition, the overall effect and the effects in some subgroups showed significant heterogeneity. In dealing with this heterogeneity, we applied the steps proposed by Petitti [34]. It seems reasonable to assume that the heterogeneity of the effects found can be attributed in part to the variance in the specific subjects included in the primary studies. Various aspects of cognitive performance were considered in the studies, for example, the effects of smartphone presence and smartphone use in different skill domains, such as mathematics, language comprehension, and spelling [14]. Another study focused on the “stroop effect” and the “switch-cost effect” [21]. For this reason, we calculated separate meta-analyses for “memory”, “attention”, and “general cognitive performance”. We again detected significant heterogeneity in each of these subgroup analyses. It is possible that the variance that continues to exist is due to the use of different measurement instruments. For example, some study designs used “Raven’s Standard Progressive Matrices Test” or the “Spanboard Test” [16], while others used a “trail making test” or a test to capture “digit cancellations” [30]. An analysis of possible moderators suggests that the nationality of the individuals studied contributes to this variance. The investigation of further possible influencing factors was omitted, because the small number of studies did not allow for the extraction of any further, theoretically significant moderators. Furthermore, not all of the studies reported potentially important personal characteristics, such as age, social origin, educational background, gender, and degree of smartphone dependence. These factors could be crucial when it comes to the distracting effect of smartphones. We recommend that further studies should address the identification and investigation of possible moderators.

Despite the heterogeneous findings, the results of the meta-analysis indicate a negative effect of smartphone use or smartphone presence on various aspects of cognitive performance. Therefore, we confirmed the tendencies found in the Brain Drain study. Our findings indicate a distracting effect of smartphones, even if they are not actively used. For this reason, two aspects of media education are important from an educational point of view [35]. On the one hand, it is necessary to protect children in particular from the use of smartphones that is not controlled in terms of content and time. To this end, it will also be necessary to discuss bans, especially in schools, as recently outlined by UNESCO in its Global Education Monitoring Report (2023) [36]. On the other hand, we need school concepts that introduce young people to the use of smartphones, with a high degree of self-reflection at the centre. This requires not only knowledge of technical aspects, but also, and above all, knowledge of the distraction potential of smartphones and their influence on memory, attention, and general cognitive performance. A clear distinction should be made between the smartphone as a work tool in the classroom and the use of the device for private purposes. The available research findings show that smartphones should not be used without reflection. Due to their distracting potential, their use should always be accompanied by a pedagogical-didactic added value, which should always be examined. We cannot support calls for smartphones to be widely used in schools with little regulation. Based on the available research findings, it seems advisable that smartphones should not even be near learners during periods of learning.

Nevertheless, smartphones are an important part of our living environment. Consequently, it is necessary to empower people, especially children and young people, to take advantage of smartphone opportunities while avoiding the dangers. The knowledge of the Brain Drain effect is essential for this.

## Figures and Tables

**Table 1:** Heterogeneity of the factors.

Factor	*Q* (df)	*I* ^2^
Memory	36.14 ** (17)	52.96
Attention	32.30 * (18)	44.29
General cognitive performance	37.37 ** (5)	86.62

Notes. *Q* = Cochran’s Q. df = degrees of freedom, *I*^2^ = according to DerSimonian and Laird. * = *p* < 0.05. ** = *p* < 0.01.

**Table 2:** Heterogeneity of effects depending on origin.

Factor	*Q* (df)	*I* ^2^
North America	48.96 ** (24)	50.98
Europe	30.73 ** (9)	70.71
Asia	10.08 (5)	30.58

Notes. *Q* = Cochran’s Q. df = degrees of freedom, *I*^2^ = according to DerSimonian and Laird. ** = *p* < 0.01.

## Data Availability

Data can be requested from the corresponding author (klaus.zierer@uni-a.de).

## Appendix A. Selected Studies with Further Information

**Author/Title**	**Region**	**Results**	**Effects**	**n**	
Domain: General cognitive performance					
Boila, V. C., Kwong, T. E., and Hintz, J. E. (2017) [18]	North America	The group with a cell phone present outperformed the group without a cell phone in all subtests (sentence comprehension, spelling, and math subtest).	Sentence comprehension subtest: phone present (*M* = 100.45, *SD *= 10.17) phone absent (*M* = 98.18, *SD *= 12.96), *t*(42) = 0.65, *p* = 0.52, *g* = 0.19, 95% CI (−4.82, 9.36) Spelling subtest: phone present (*M* = 106.71, *SD *= 6.80) phone absent (*M* = 102.35, *SD *= 8.09), *t*(39) = 1.87, *p* = 0.07, *g* = 0.58, 95% CI (−0.35, 9.08) Mathematics subtest: phone present (*M* = 92.64, *SD *= 9.05), phone absent (*M* = 88.32, *SD *= 8.49), *t*(42) = 1.63, *p* = 0.11, *g* = 0.49, 95% CI (−1.02, 9.66)	45	
Stone, J. (2020) [8]	North America	Cell phone presence inhibited performance. Cell phone visibility reduces cognitive performance.	Fluid intelligence: desk condition (*M* = 63.28, *SD *= 9.10, *n* = 19) other room (*M* = 51.55, *SD *= 12.13, *n* = 18) *t*(35) = 3.312, *p* = 0.002	37	
Toader, J. F. et al. (2022) [29]	Europe	The data presented show that distraction by phone calls decreases the performance level of medical students during an OSCE station.	OSCE I (students with both stations *n* = 13 each): not distracted by phone calls (*M* = 8.48, *SD *= 0.89) distracted by telephone calls (*M* = 7.39, *SD *= 0.91) OSCE II (students with both wards, *n* = 101 each): not distracted by phone calls (*M* = 7.61, *SD *= 1.16) distracted by telephone calls (*M* = 6.92, *SD *= 1.23)	308	
Domain: Memory					
Nakagawa, N. et al. (2022) [23]	Asia	The presence of the smartphone did not affect electroencephalography results or working memory.	*g* = −0.25, 95% CI = −0.58, 0.09	36	
Stone, J. (2020) [8]	North America	Results showed that cell phone presence inhibited performance. Cell phone visibility reduces cognitive performance.	Working memory capacity: other room (*M* = 64.24, *SD* = 6.05, *n* = 18) desk condition (*M* = 57.80, *SD* = 7.08, *n* = 19); *t*(35) = 2.965, *p* = 0.005	37	
Quanbrough, J. (2018) [25]	Europe	The results showed that smartphone presence impaired the participants’ cognitive performance.	Working memory capacity: smartphones present (*n* = 51, *M* = 10.43, *M* = 11.00, *SD* = 3.00) smartphones absent (*n* = 48, *M* = 11.66, M = 12.00, *SD* = 2.89) (*W* = 1464.5, *p* = 0.03, *r* = −0.22, *g* = 0.42)	99	
Tanil, C. T. and Yong, M. H. (2020) [27]	North America	Presence of a smartphone and high phone awareness negatively affect memory learning and recall.	Presence (HS) or absence (LS) of smartphone. LS (*M* = 14.21, *SD* = 2.61), HS (*M* = 13.08, *SD* = 2.53), *t*(117) = 2.38, *p* = 0.02, *g* = 0.44	119	
Hartmann, M., Martarelli, C. S., Reber, T. P., and Rothen, N. (2020) [20]	Europe	No overall effect of smartphone presence on short-term and perspective memory performance. Better performance in participants with low smartphone dependence when smartphone was not present.	F(1, 300) = 0.17, *p* = 0.676	302	
Hartanto, A. and Yang, H. (2016) [21]	Asia	Smartphone absence leads to reduction in working memory capacity. Impairment of mental shifting independent of the extent of smartphone addiction.	Stroop task (assesses inhibitory-control processing): effect of smartphone separation: *B* = 47.23, *p* = 0.028 Rotation span test (working-memory capacity): effect of smartphone separation: *t*(60) = 1.98, *p* = 0.052, *g* = 0.52	70	
Ward, A. F., Duke, K., Gneezy, A., and Bos, M. W. (2017) [3]	North America	The mere presence of smartphones can negatively affect the available capacity of working memory.	Effect 1: *g* = −0.17, 95% CI = −0.47, 0.12 Effect 2: *g* = −0.25, 95% CI = −0.58, 0.09	521	
Niu, G. et al. (2022) [24]	Asia	The presence of smartphones has a negative impact on cognitive functions.	*g* = −0.29, 95% CI = −0.68, 0.10	100	
Canale, N., Vieno, A., Doro, M., Rosa Mineo, E., Marino, C., and Billieux, J. (2019) [22]	Europe	Turned-on devices had a detrimental effect on subjects’ task performance.	Effect 1: *g* = −0.29, 95% CI = −0.73, 0.15 Effect 2: *g* = −0.19, 95% CI = −0.25, 0.63	159	
Ruiz Pardo, A., and Minda, J. (2022) **[18]**	North America	The mere presence of the smartphone is not enough to affect cognitive performance.	Effect 1: *g* = −0.17, 95% CI = −0.52, 0.19 Effect 2: *g* = −0.08, 95% CI = −0.42, 0.25 Effect 3: *g* = −0.03, 95% CI = −0.38, 0.33 Effect 4: *g* = 0.14, 95% CI = −0.21, 0.49	511	
Domain: Attention					
Aguila, B. (2019) [31]	North America	Presence of a cell phone led to poorer performance among participants with smartphones in close proximity.	*g* = −0.71, 95% CI = −1.30, −0.12	57	
Ruiz Pardo, A., and Minda, J. (2022) [18]	North America	The mere presence of the smartphone is not enough to affect cognitive performance.	Effect 5: *g* = 0.00, 95% CI = −0.33, 0.33 Effect 6: *g* = 0.00, 95% CI = −0.35, 0.35 Effect 7: *g* = 0.13, 95% CI = −0.22, 0.49 Effect 8: *g* = 0.13, 95% CI = −0.22, 0.49	511	
Moshiri, J. (2018) [17]	North America	Smartphone presence had no significant effect on cognitive performance.	Effect 1: *g* = 0.05, 95% CI = −0.35, 0.45 Effect 2: *g* = 0.16, 95% CI = −0.24, 0.56	192	
Ward, A. F., Duke, K., Gneezy, A. and Bos, M. W. (2017) [3]	North America	Presence of smartphone can tax cognitive resources, leaving fewer resources available for other tasks and impairing cognitive performance.	Effect 3: *g* = 0.07, 95% CI = −0.22, 0.36 Effect 4: *g* = 0.07, 95% CI = −0.22, 0.37	361	
Koessmeier, C. and Büttner, O. (2022) [19]	Europe	Presence of the smartphone without influence on task performance.	*g* = 0.10, 95% CI = −0.32, 0.53	86	
Lyngs, U. (2017) [15]	Europe	Presence of smartphone without influence on task performance.	*g* = 0.20, 95% CI = −0.34, 0.74	53	
Thornton, B., Faires, A., Robbins, M., and Rollins, E. (2014) [30]	North America	Mere presence of a cell phone sufficiently distracting to lead to decreased attention and deficits in task performance, especially in tasks with higher attentional and cognitive demands.	Digit cancellations: cell phone present (experimental group, *M* = 21.29) no cell phone present (*M* = 26.17) *F*(1, 45) = 5.80, *p* < 0.05, *g2 p* = 0.11 Trail making tests: more difficult Part B: phone present (*M* = 14.50) phone not present (*M* = 16.91) *F*(1, 45) = 4.05, *p* = 0.05, *g2 p* = 0.08	54	
Gutierrez-Puertas et al. (2020) [26]	Europe	More attentive in class without access to cell phones.	Mindful attention awareness scale: smartphone absent (*M* = 54.17, *SD* = 14.30, *n* = 63) smartphone present, *M* = 48.27, *SD* = 12.71, *n* = 61)	124	
Mahsud, M., Khaaf, A. J. M., Mahsud, Z., Afzal, A., Afzal, F. (2021) [9]	Asia/Europe	Concentration disturbed by smartphones. Asian concentrate better without smartphones. European students became restless without smartphones.	European students (*n* = 18): without smartphone: *M* = 4.7, *SD* = 3.34 with smartphone: *M* = 8.25, *SD* = 1.92 Asian students (*n* = 22): without smartphone: *M* = 5, *SD* = 2.44 with smartphone: *M* = 5.25, *SD* = 8.02	40	
Stavrum, M. (2020) [16]	North America	No statistically significant effect of smartphone availability on performance.	Raven’s Standard Progressive Matrices: smartphone present (*M* = 27.45, *SD* = 1.78) smartphone absent (*M* = 27.15, *SD* = 1.88) Spanboard Test: smartphone present (*M* = 6.6, *SD* = 1.78) smartphone absent (*M* = 6.5, *SD* = 1.39)	40	
Mendoza, J. S., Pody, B. C., Lee, S., Kim, M., and McDonough, I. M. (2018) [28]	North America	Participants distracted by the smartphone performed worse in the test than those who were not distracted.	Main effect of group was found: *F*(1158) = 7.51, *MSE* = 0.029, *p* = 0.007, *h*^2^*p* = 0.05 Quiz performance: cellphones removed (*M* = 0.65, *SD* = 0.17) cellphones retained (*M* = 0.57, *SD* = 0.16)	160	
Note. n = number of observations.		
